# Sponge holobionts shift their prokaryotic communities and antimicrobial activity from shallow to lower mesophotic depths

**DOI:** 10.1007/s10482-022-01770-4

**Published:** 2022-08-23

**Authors:** Anak Agung Gede Indraningrat, Georg Steinert, Leontine E. Becking, Benjamin Mueller, Jasper M. de Goeij, Hauke Smidt, Detmer Sipkema

**Affiliations:** 1 Laboratory of Microbiology, Wageningen University and Research, Stippeneng 4, 6708 WE Wageningen, The Netherlands; 2 Faculty of Medicine and Health Sciences, Warmadewa University, Jln Terompong 24, 80235 Denpasar, Bali, Indonesia; 3 Marine Animal Ecology Group, Wageningen University and Research, Droevendaalsesteeg 1, 6708 PB Wageningen, The Netherlands; 4 Wageningen Marine Research, Wageningen University and Research, Ankerpark 27, 1781 AG Den Helder, The Netherlands; 5 Department of Freshwater and Marine Ecology, University of Amsterdam, P.O. Box 94240, 1090 GE Amsterdam, The Netherlands; 6 CARMABI Foundation, Piscaderabaai z/n, P.O. Box 2090, Willemstad, Curaçao

**Keywords:** Sponges, Prokaryotic community, Depth, Antimicrobial activity

## Abstract

In this study, we used 16S rRNA gene amplicon sequencing to investigate prokaryotic community composition of the Caribbean sponges *Xestospongia muta* and *Agelas sventres* from three depth ranges: < 30 m (shallow), 30–60 m (upper mesophotic), and 60–90 m (lower mesophotic). The prokaryotic community in shallow samples of *X. muta* was enriched in Cyanobacteria, Chloroflexota, and Crenarchaeota compared to samples from mesophotic depths, while mesophotic samples of *X. muta* were enriched in Acidobacteriota. For *A. sventres*, relative abundance of Acidobacteriota, Chloroflexota, and Gammaproteobacteria was higher in shallow samples, while Proteobacteria and Crenarchaeota were enriched in mesophotic *A. sventres* samples. Antimicrobial activity was evaluated by screening crude extracts of sponges against a set of Gram-positive and Gram-negative bacteria, a yeast, and an oomycete. Antibacterial activities from crude extracts of shallow sponge individuals were generally higher than observed from mesophotic individuals, that showed limited or no antibacterial activities. Conversely, the highest anti-oomycete activity was found from crude extracts of *X. muta* individuals from lower mesophotic depth, but without a clear pattern across the depth gradient. These results indicate that sponge-associated prokaryotic communities and the antimicrobial activity of sponges change within species across a depth gradient from shallow to mesophotic depth.

**Supplementary Information:**

The online version contains supplementary material available at 10.1007/s10482-022-01770-4.

## Introduction

Sponges (phylum Porifera) occupy a wide range of habitats from shallow-water to deep-sea ecosystems and from tropical to polar regions (Downey et al. 2012; Van Soest et al. 2012). In these habitats, sponges fulfil prominent ecosystem functions, such as seafloor structuring, involvement in various biogeochemical cycles, and the provision of shelter for other marine fauna (Bell 2008; De Goeij et al. 2017; Beazley et al. 2013). Sponges are commonly associated with a wide variety of microbial taxa (e.g., bacteria, archaea, eukaryotes) that live within their tissues. Generally, they can be classified as either low microbial abundance (LMA) or high microbial abundance (HMA) sponges (Hentschel et al. 2003). LMA sponges contain microbial communities similar in concentration to the ambient seawater, whereas HMA sponges can host up to four orders of magnitude higher microbial concentrations, which may constitute more than one-third of the sponge holobiont’s biomass (Hentschel et al. 2003). Moreover, these communities are generally distinct from those present in the surrounding seawater and remain stable through space and time (Erwin et al. 2012, 2015; Pita et al. 2013; Hardoim and Costa 2014; Gantt et al. 2017; Enticknap et al. 2006), indicating their specificity for co-habitation with sponges (Schmitt et al. 2012; Simister et al. 2012a; Thomas et al. 2016) Sponge-associated microbial communities are considered to play important roles in nutrient cycles within the host tissue (Keren et al. 2017; Mohamed et al. 2010; Taylor et al. 2007; Zhang et al. 2015) as well as in defence mechanisms through the production of bioactive secondary metabolites (Hentschel et al. 2012; Horn et al. 2016; Slaby et al. 2017). To date, more than 11,000 of these sponge-derived compounds have been described and many of these compounds display unique features which are potentially applicable to therapeutic uses (MarinLit, 2022).

However, ecological and biotechnological studies on sponges and their associated microbial communities are typically performed in easily accessible, shallow-water (< 30 m) depth habitats, for example on tropical coral reefs. Comparable information from deeper parts of those reefs, the so-called mesophotic zone (30–150 m water depth), (Lesser et al. 2018, 2009; Kahng et al. 2010; Slattery et al. 2011), or a depth gradient are largely unavailable due to more challenging logistics (Morrow et al. 2016; Olson and Gao 2013; Steinert et al. 2016). Mesophotic coral reefs are deeper reef communities whose community structure and function change with increasing depth based on the availability of light and trophic resources (Lesser et al. 2018). In particular, primary producers, such as corals and macroalgae decrease in abundance with increasing depth due to light limitation, while sponges were found to increase in abundance (Lesser et al. 2020, 2018). Mesophotic communities are commonly further divided in upper mesophotic (30–60 m) and lower mesophotic zone (60–150 m) (Lesser et al. 2018).

From a biotechnological perspective, especially the functional diversity of secondary metabolism-associated gene clusters in mesophotic habitats and beyond is largely underexplored and may harbour different sources of novel compounds for therapeutic and industrial applications (Sipkema 2017). For example, unique polyketide synthase and non-ribosomal peptide synthetase gene clusters of microbial origin were reported from three deep-sea sponges *Inflatella pellicula, Poecillastra compressa*, and *Stelletta normani*, which may provide hints to novel compounds (Borchert et al. 2016). Furthermore, the TARA Ocean study highlighted the sharp increase of unknown functional genes as depth increased (Sunagawa et al. 2015), which further reinforces the potential of bioprospecting for novel compounds in mesophotic habitats and beyond (Sipkema 2017). Whether similar patterns can be observed across a narrower depth gradient from shallow to mesophotic depths needs to be explored. Differences in the environmental conditions between shallow water and the mesophotic zone relate to light intensity, temperature, nutrient availability, predation, and human impact. These altered (a)biotic factors may lead to differences in microbial community composition in marine invertebrate holobionts (i.e. host and symbionts), including sponges (Morrow et al. 2016; Olson and Gao 2013; Slattery et al. 2016; Steinert et al. 2016). Accordingly, these studies indicate that while sponges may maintain a stable core of associated bacteria from shallow water to mesophotic depth, certain sponge-associated bacterial taxa vary with depth (Olson and Gao 2013). A study from the Pacific indicated a significant change of the prokaryotic community composition of the sponge *Callyspongia* sp. From shallow to mesophotic habitats. However, the exact environmental factor(s) responsible for such differences could not be determined (Steinert et al. 2016). Additionally, more comprehensive studies on coral holobionts have indicated morphological adaptations and symbiont specializations with greater depth, with species inhabiting the mesophotic zone harbouring a specific photosynthetic endosymbiont (*Symbiodinium*) community to adapt to low light conditions (Bongaerts et al. 2015; Brazeau et al. 2013; Gonzalez-Zapata et al. 2018; Lesser et al. 2010; Vermeij and Bak 2002).

Sponges commonly use secondary metabolites as chemical defence to deter predation and fouling and to compete for space with neighbouring benthic marine organisms (Pawlik et al. 1995; Loh and Pawlik 2014; Page et al. 2005). In some cases, these compounds also serve as general antimicrobial substances (Webster 2007; Newbold et al. 1999; Sarah et al. 2003). However, little is known about how the metabolomes of sponges change over depth and first studies are rather opposing. While the transplantation of *Aplysina cavernicola* from 40 m to 7–15 m did not alter its metabolite profile (Thoms et al. 2003), the transplantation of *Plakortis angulospiculatus* from 10 to 75 m and vice versa resulted in a much stronger deterrent effect of shallow-adapted individuals towards predation by the spongivorous pufferfish *Canthigaster rostrata* compared to deep-adapted specimens (Slattery et al. 2016).

To further elucidate the effect of water depth on prokaryotic community composition as well as the production and antimicrobial activity of secondary metabolites we studied two Caribbean HMA sponge species—*Xestospongia muta* and *Agelas sventres*—that commonly occur across the entire shallow-to-lower mesophotic depth gradient. Moreover, both species have been reported to produce bioactive compounds. In *X. muta*, multiple secondary metabolites with predator-deterrent and antimicrobial activities were identified (Chanas and Pawlik 1997; Morinaka et al. 2007; Patil et al. 1992), whereas only one bioactive compound, the feeding-deterrent compound sventrin, has been reported in *A. sventres* (Assmann et al. 2001). We sampled both species at three depths: < 30 m (shallow), 30–60 m (upper mesophotic depth) and 60–90 m (lower mesophotic depth). For all samples (1) the prokaryotic community composition was determined using Illumina MiSeq 16S rRNA gene amplicon sequencing, (2) the antimicrobial activity of sponge tissue extracts was examined against six microbial indicator strains—two Gram-positive and two Gram-negative bacterial strains, a yeast and an oomycete.

## Material and methods

### Sample collection and sponge tissue processing

*Xestospongia muta* and *Agelas sventres* individuals were collected between 4 and 22 November, 2015 on the reef slope in front of the Substation (12°05′04.4″N 68°53′53.7″W) on the leeward side of Curaçao, Southern Caribbean. Samples were collected from three different depths along a shallow-mesophotic depth gradient: < 30 m (shallow), 30–60 m (upper mesophotic), and 60–90 m lower mesophotic) (bottom of Fig. 2). From each depth, five biological replicates (i.e. different individual sponges; *n* = 5) were collected for each species. Shallow sponge individuals were collected by SCUBA diving, while upper and lower mesophotic individuals were taken using a submarine, the “Curasub”. Three 1-L seawater samples were collected from each depth using a Niskin bottle to serve as background seawater prokaryotic community profile. Upon arrival in the laboratory, sponges were cleaned from visible debris (e.g. mud, sand), rinsed three times using sterile artificial seawater (ASW, 33 g L^−1^ synthetic sea salt [Instant Ocean Reef Crystals, Aquarium Systems, Sarrebourg, France]) and were cut into pieces of ~ 0.1 cm^3^. Three to four randomly chosen pieces of tissue from each individual were preserved in a 15 mL Falcon tube (Sigma-Aldrich) containing 10 mL of RNA*later* stabilization solution (Thermo Fisher Scientific). Seawater samples were filtered through 0.2-µm pore size nitrocellulose filters (Sigma-Aldrich). The preserved sponge tissues and filters were stored at − 20 °C until further analysis.

### DNA extraction

DNA was extracted from sponge samples (~ 200 mg biomass per sample) and seawater filters using the Fast DNA Spin kit for soil (MP biomedicals) following manufacturer’s instructions with the slight modification by conducting 2 times 45 s of bead beating cell lysis (Precellys 24 Bertin Instruments, Montigny-le-Bretonneux, France). DNA concentrations were checked using a spectrophotometer (DeNovix DS-11, Wilmington, USA) and the quality of DNA was visualized on a 1% agarose gel.

### Sponge identification

Sponge specimens were identified by manually inspecting the type of spicules of each specimen. Furthermore, molecular identification of sponge samples was conducted by amplifying cytochrome oxidase subunit 1 (COI) encoding genes using primers dgLCO1490F (5′-GGT CAA CAA ATC ATA AAG AYA TYG G-3′) and dgHCO2198R (5′-TAA ACT TCA GGG TGA CCA AAR AAY CA-3′) (Meyer et al. 2005). PCR amplification of the COI fragment was performed in a volume of 50 µL containing 28.75 µL nuclease free water, 10 µL 5 × Green Gotaq Flexi buffer, 1 µL 10 mM dNTPs, 1 µL forward primer (10 µM), 1 µL reverse primer (10 µM), 3µL MgCl_2_ (25 mM), 4 µL Bovine Serum Albumin (BSA), 0.25 µL Gotaq HotStart DNA Polymerase (5 U/µL) and 1 µL DNA (10–20 ng), following the protocol as previously described (Meyer et al. 2005). PCR products were visualised on a 1% agarose gel, purified using the Thermo Scientific GeneJET PCR Purification Kit and Sanger sequenced in both directions (GATC Biotech AG, Germany). The chromatograms of forward and reverse COI sequences of each specimen were assembled and quality checked manually using Geneious (Kearse et al. 2012) version 10.0.9. Additionally, six and four reference COI sequences of *X. muta* and *A. sventres*, respectively, were retrieved from the Sponge Gene Tree server (Erpenbeck et al. 2008), along with COI sequences of other sponge species as outgroups. All COI sequences were aligned using MEGA6 (Tamura et al. 2013) with the MUSCLE algorithm resulting in a final sequence length of 644 nt and 707 nt for *X. muta* and *A. sventres*, respectively. Subsequently, phylogenetic trees were generated in MEGA6 based on the COI sequences by applying the maximum likelihood algorithm (Tamura et al. 2011) with 500 bootstrap replicates and the Nearest-Neighbor-Interchange (NNI) to optimise tree topology.

### Prokaryotic community profiling using 16S rRNA gene amplicon sequencing

Prokaryotic community composition was assessed by Illumina MiSeq amplicon sequencing of the V4 region of the 16S rRNA gene using a two-step amplification procedure. PCR was conducted using the 2^nd^ version of the EMP (Earth Microbiome Project) primer pair 515F (5′GTGYCAGCMGCCGCGGTAA3′) (Parada et al. 2016) and 806R (5′GGACTACNVGGGTWTCTAAT3′) (Apprill et al. 2015). Subsequently, Unitag 1 and Unitag 2 were added to the forward and reverse primer, respectively, as previously described (Van Lingen et al. 2017). In the first step PCR, 25 μL PCR reactions contained 16.55 μL nuclease free water (Promega, Madison, USA), 5 μL of 5 × HF buffer, 0.2 μL of 2 U/μL Phusion hot start II high fidelity polymerase (Thermo Fisher Scientific AG), 0.75 μl of 10 μM stock solutions of each primer, 0.75 μL 10 mM dNTPs (Promega) and 1 μL DNA (10–20 ng). Amplification was performed at 98 °C for 3 min, followed by 25 cycles at 98 °C for 25 s, 50 °C for 20 s, 72 °C for 20 s and a final extension of 7 min at 72 °C. PCR products were visualized on a 1% (w/v) agarose gel. Subsequently, 5 μL of these first-step PCR products were used as template in the second PCR reaction to incorporate 8 nt sample specific barcodes. The second step PCR was performed in triplicate for each sample in 50 μL PCR reactions which contained 31 μL nuclease free water (Promega), 10 μL of 5 × HF buffer, 0.5 μL of 2 U/μL Phusion hot start II high fidelity polymerase (Thermo Fisher Scientific AG), 5 μL equimolar mixes of 10 µM forward primer (barcode-linker-Unitag1) and reverse primer (barcode-linker-Unitag2), 1 μL 10 mM dNTPs (Promega) and 2.5 µL of the first PCR product as template. The second step PCR was performed for five cycles with the same amplification program as the first step PCR. The PCR products were purified following a method as previously described (Dat et al. 2018), and the purified library was sequenced at GATC Biotech AG (Germany) by Illumina Miseq sequencing.

### Raw sequence processing

Raw sequence data was processed using a previously described protocol (Dat et al. 2018) with slight modifications. Specifically, raw data was analyzed using NG-Tax (Galaxy version 1.0) (Ramiro-Garcia et al. 2016) with forward and reverse paired-end reads being trimmed to 70 nucleotides. NG-Tax is an open sequencing platform for high-throughput 16S rRNA gene amplicon analysis and has been applied to detect prokaryotic composition from different niches (Wampach et al. 2018; Deng et al. 2021; Dat et al. 2018; Edwards et al. 2020). Subsequently, both reads were concatenated, resulting in sequences of 140 bp as an optimum accurate length that was used for further sequence data processing (Poncheewin et al. 2020). Taxonomic assignment was done by utilizing a customized version of the SILVA 138 SSU database (Yilmaz et al. 2014), and ASVs classified as Chloroplasts and Mitochondria were removed from the analysis.

### Prokaryotic community analysis

Data analyses were performed in R version 3.5.0 (https://www.r-project.org) and Microsoft Excel. Community 16S rRNA gene abundance data processing and analyses in R were performed using the following R packages: phyloseq version 1.21.0 (Mcmurdie and Holmes 2013), microbiome version 0.99.90 (Lahti et al. 2017), and ggplot2 version 2.2.1 (Wickham 2016). The NG-Tax generated phylogenetic ASV tree was processed using the ape package version 4.1 (Paradis et al. 2004), and phylogenetic diversity was calculated using the picante package version 1.6-2 (Kembel et al. 2010). Phylogenetic diversity of each group of samples was analyzed using Kruskal–Wallis and Wilcoxon rank sum test to assess significance of potential differences among groups of samples for the parameters “sample types” (i.e. *X. muta*, *A. sventres*, and seawater) and “depth” (i.e. shallow, upper mesophotic, lower mesophotic). The raw *p*-values were adjusted using the Benjamin-Hochberg method (Benjamini and Hochberg 1995). The prokaryotic community composition was visualized by principal coordinate analysis (PCoA) based on Hellinger transformed relative abundances of ASVs using Bray–Curtis distances. The adonis and betadisper functions as implemented in vegan package version 2.5.2 (Kolde 2015) were employed to estimate the variance and dispersion of beta diversity, by applying two factors: “sample type” and “depth”.

A heatmap was generated in R using pheatmap version 1.0.8 (Kolde 2015) for the most abundant ASVs (≥ 0.25%, *n* = 100) based on average relative abundance across all samples. Subsequently, the most abundant ASVs listed in the heatmap (*n* = 100) were used to identify ASVs that were significantly enriched in the 3569 sponge specimens (comprising 269 sponge species) from the sponge microbiome project (Moitinho-Silva et al. 2017). Sequence comparison was done based on a method described previously (Dat et al. 2018). Briefly, sponge microbiome project subOTU sequences were selected based on having no more than one nucleotide mismatch with sequences of the most abundant ASVs observed in this study. The selected subOTU sequences were then uploaded to the spongeEMP online server (www.spongeemp.com) to identify ASVs that were significantly enriched in sponges. Furthermore, the most abundant ASVs were checked by a G-test using the script *group_significance.py* in QIIME version 1.9.1, and raw *p*-values were adjusted using the Benjamin-Hochberg FDR correction for multiple comparisons.

### Preparation of crude extract of sponge tissue and antimicrobial activity screening

Crude extracts of sponge tissues were prepared based on a previous method (Rohde et al. 2015) with a slight modification on the amount of starting tissue samples. Briefly, 0.3 g of lyophilized sponge sample was transferred to a 35 mL glass tube (Kimax) and resuspended in 10 mL methanol:ethyl acetate (1:1). The tube was incubated at room temperature (20 °C) and shaken at 150 rpm for 20 min, followed by 10 min of centrifugation at 427*g* (Thermo Scientific Sorvall Legend XTR Centrifuge TX-1000, Waltham, Massachusetts). Crude extracts were transferred into pre-weighed glass tubes and evaporated to dryness with a speed-vac (Eppendorf Vacufuge Concentrator, Hamburg, Germany). Extraction of each sponge sample was conducted three times, and the crude extracts obtained from each of the extractions were pooled in the same pre-weighed glass tube and stored at − 20 °C until further use.

Six microbes were used as indicator strains to evaluate antimicrobial activity of sponge extracts, namely the Gram-positive bacteria *Bacillus subtilis* DSM 402 and *Staphylococcus simulans* DSM 20037, Gram-negative bacteria *Escherichia coli* K12MG1655 and *Aeromonas salmonicida* DSM 19634, the yeast *Candida oleophila* DSM 70763, and the oomycete *Saprolegnia parasitica* CBS223.65. These indicators were selected because they represent causative agents of diseases in animals. Briefly, the following growth media were used for the bacterial and yeast strains: liquid Lysogeny Broth medium (LB, Oxoid) for *E. coli*, Nutrient broth (Oxoid) for *A. salmonicida* and *B. subtilis*, Trypticase Soy Yeast Extract for *S. simulans* (DSMZ medium no. 92) and Universal Medium for Yeast (DSMZ medium no. 186) for *C. oleophila*. Cultures were grown until an optical density of 0.5 was reached, measured at 660 nm. Subsequently, 200 µL of each active culture was spread with a sterile hockey stick on agar media with the same composition as the corresponding liquid media. The oomycete *S. parasitica* was prepared by inoculating agar plugs of 1 × 1 cm from a lawn of fresh *S. parasitica* culture plate on one-fifth strength of Potato Dextrose Agar (PDA, Oxoid) plates supplemented with 1% of Bacto agar (Oxoid).

Antimicrobial properties of each crude extract were examined using the disc diffusion assay (Rohde et al. 2015) by adding 20 µL extract (0.5 mg per disc) to three 6 mm cellulose paper discs (Whatman). Paper discs containing the crude extract were air-dried for 30 min. As negative control, triplicate discs containing 20 µl methanol: ethyl acetate (1:1) were included. Paper discs containing sponge crude extracts were tested against indicator strains on agar plates. Plates containing sponge extracts and indicator strains were incubated at 37 °C for *E. coli* and *S. simulans*, at 30 °C for *A. salmonicida, B. subtilis* and at 20 °C for *C. oleophila* for 48 h. The plates containing extracts and *S. parasitica* were incubated at 20 °C for 96 h. After incubation, the radius of the zone of inhibition (ZOI) surrounding each disc was measured to the nearest mm using digital callipers (Perel, Gavere, Belgium), and the average ZOI radius for each extract was calculated from triplicate discs. In addition, to differentiate the level of inhibition from each crude extract, the recorded ZOI radius was grouped into four categories: weak (0–5 mm), moderate (6–10 mm), strong (10–20 mm) and very strong (> 20 mm) (Davis and Stout 1971). Analysis of variance (ANOVA) was done to compare average ZOIs formed by sponge crude extracts against indicator strains. Subsequently Tukey Post-Hoc test was applied to assess significance in ANOVA.

## Results

### Molecular sponge taxonomy

Genetic analysis of the COI gene sequences obtained from all sponge samples used for this study showed that all COI sequences of suspected *Xestospongia muta* samples could be assigned to the same species (Supplementary Fig. 1A). In contrast, four out of five of the suspected *Agelas sventres* samples from the lower mesophotic depth formed a separate clade from the other *A. sventres* samples and *A. sventres* reference COI gene sequences. These four lower mesophotic samples may therefore either represent a new undescribed or another known *Agelas* species for which no COI gene sequence is available (Supplementary Fig. 1B). Hence, all *Agelas* samples from the lower mesophotic depth were excluded from further analysis as taxonomy could not be unequivocally established.

### Impact of water depth on sponge-associated prokaryotic communities

Across 34 samples (25 sponge and 9 seawater samples), 2,277,222 high quality reads were clustered into 4394 Amplicon Sequence Variants (ASVs) (Table 1; Supplementary Table 1). *X. muta* samples yielded the highest number of observed ASVs on average, followed by seawater and *A. sventres* samples (Supplementary Fig. 2).

**Table 1:** Overview of sample data with sample identifiers (ID), sponge species, average actual depth, and depth, average number of reads, average number of ASVs, and average phylogenetic diversity

Sample ID	Sample type	Depth	Average number of reads	Average number of ASVs	Average Phylogenetic Diversity (PD)
XM1˗XM5	*X. muta*	LM	53,595 ± 51,996	174 ± 18	17.54 ± 1.11
XM6˗XM10	*X. muta*	UM	54,253 ± 61,518	179 ± 10	18.28 ± 0.24
XM11˗XM15	*X. muta*	shallow	89,129 ± 69,868	163 ± 14	17.72 ± 0.57
AS1˗AS5	*A. sventres*	UM	63,389 ± 30,710	70 ± 6	11.94 ± 0.86
AS6˗AS10	*A. sventres*	shallow	74,756 ± 52,091	70 ± 6	12.38 ± 0.53
SW1˗SW3	Seawater	LM	93,275 ± 35,371	102 ± 50	12.74 ± 2.78
SW4˗SW6	Seawater	UM	48,825 ± 17,635	148 ± 14	15.36 ± 0.43
SW7˗SW9	Seawater	shallow	58,438 ± 8580	123 ± 5	14.52 ± 0.30

All values are given with their corresponding standard deviation. A more detailed description of individual specimens is available in Supplementary Table 1. LM: lower mesophotic; UM: upper mesophotic

**Fig. 1:**
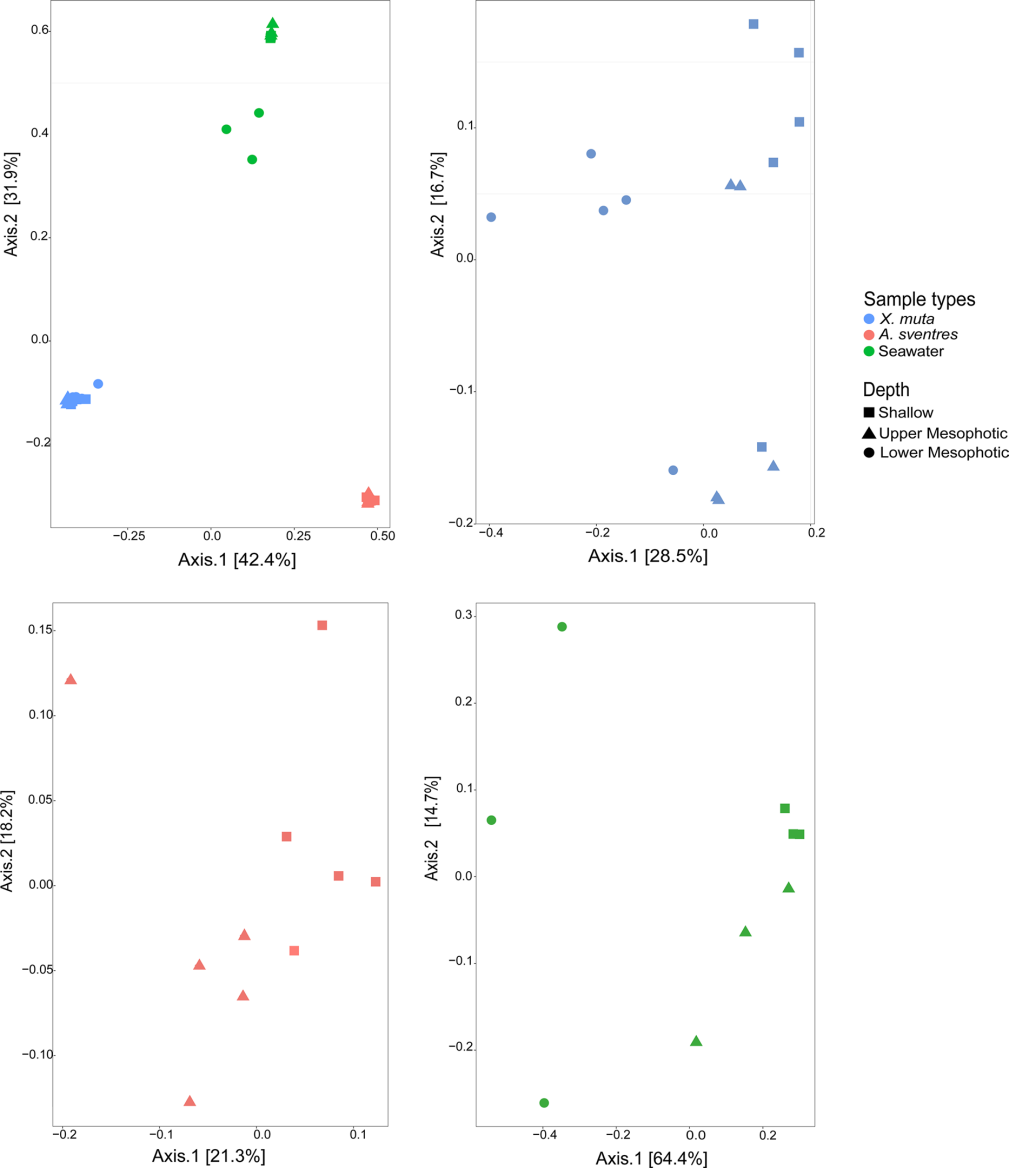
Principal coordinate analysis (PCoA) of prokaryotic community composition of **A** sponges and seawater samples using Bray–Curtis distance based on relative abundance of ASVs after Hellinger transformation. Additionally, the PCoA was done separately for each sample type: **B** *Xestospongia muta*, **C** *Agelas sventres* and **D** seawater

The prokaryotic community significantly differed among sample types (*X. muta*, *A. sventres* and seawater) (PERMANOVA, *p* = 0.001) and sample type contributed to 74% of the variance of the prokaryotic community composition (Fig. 1A; Table 2; Supplementary Table 2). Post-hoc pairwise comparison showed that both sponges significantly differed in prokaryotic community composition from seawater and from each other (*p* = 0.003 for both comparisons; Supplementary Table 3). Depth did not have a significant effect when all sponge and water samples were analysed together and contributed only 9% to the variance of the prokaryotic community composition (Table 2). However, depth did show a significant effect when the impact on prokaryotic community was analysed per sample type, contributing to 32, 18 and 66% of the variance for *X. muta*, *A. sventres*, and seawater, respectively (*p* = 0.001, 0.009, and 0.003 respectively; Table 2). Post-hoc pairwise comparison showed that for *X. muta* the prokaryotic community significantly changed between lower mesophotic depth and both the upper mesophotic and shallow depth (*p* = 0.02), but not between the upper mesophotic and shallow depth (Supplementary Table 3; Fig. 1B). Within seawater samples, there was a marked significant difference in the prokaryotic community composition between the lower mesophotic depth and the upper mesophotic and shallow depth (*p* = 0.001; Fig. 1D; Supplementary Table 3). In turn, the upper mesophotic and shallow depth did not show a significantly different prokaryotic community composition.

**Table 2:** Multivariate analysis of prokaryotic community data after Hellinger transformation based on parameter sample type (sponge and seawater), depth (sponge and seawater) and depth for subsets *Xestospongia muta*, *Agelas sventres* and seawater. A *p* value ≤ 0.05 is considered significant

Parameter	ASVs	Df	PERMANOVA		Betadisper	
			*R*^2^	*p* value	*F*	*p* value
Sample types (sponges and sea water)	4394	2	0.74	**0.001**	4.46	**0.02**
Depth (sponges and sea water)	4394	2	0.09	0.17	1.02	0.4
Depth (*X. muta* only)	2576	2	0.32	**0.001**	1.89	0.2
Depth (*A. sventres* only)	699	1	0.18	**0.009**	0.35	0.54
Depth (seawater only)	1119	2	0.66	**0.003**	7.19	**0.006**

*Df* degrees of freedom

**Fig. 2:**
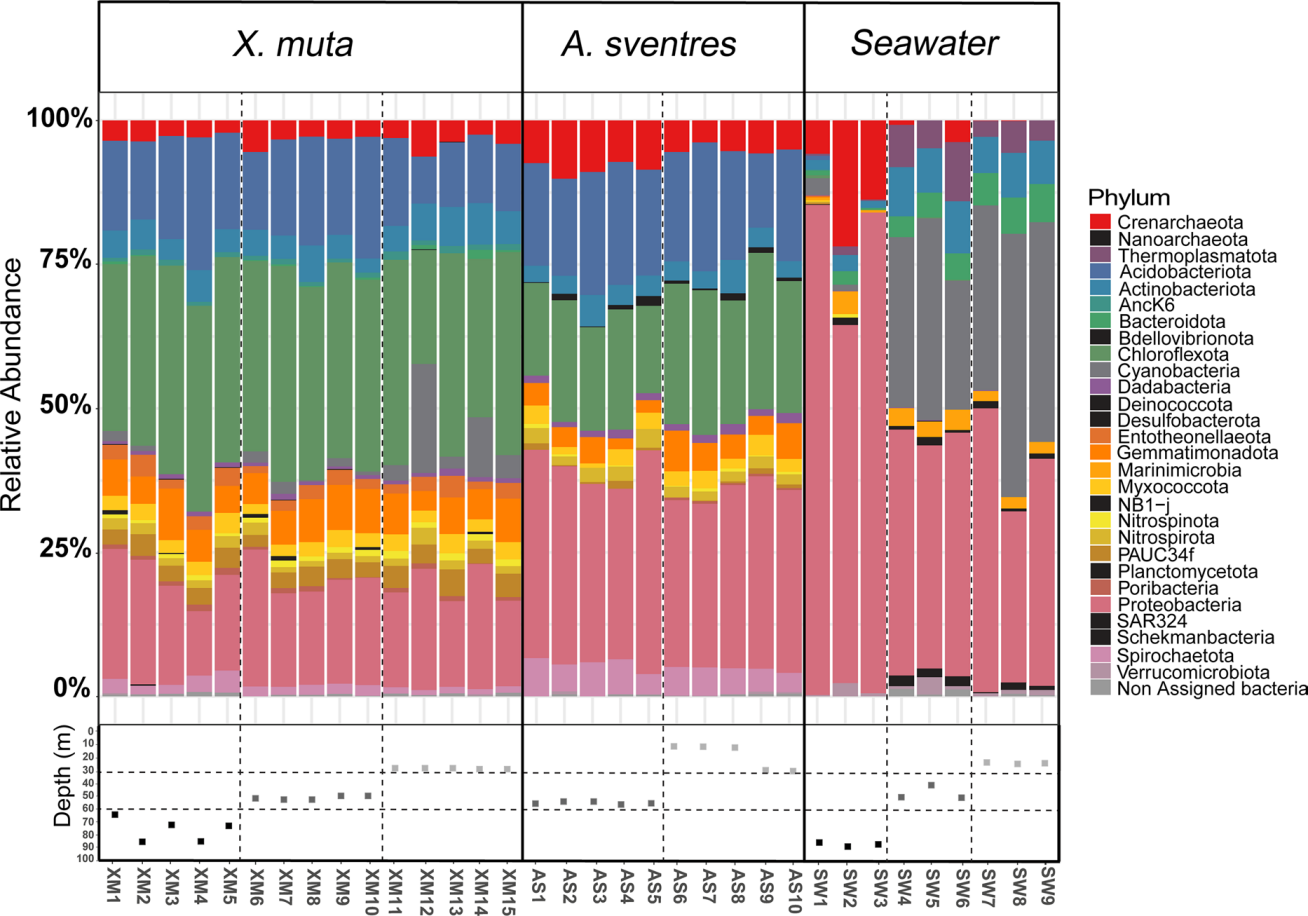
Prokaryotic community composition of sponge specimens and seawater samples at the phylum level. Phyla with average relative abundance lower than 0.25% in all samples (Bdellovibrionota, SAR324, Planctomycetota, NB1-j, Deinococcota, Schekmanbacteria, Nanoarchaeota, Desulfobacterota) were coloured in black. Sampling depth of each sponge specimen and seawater sample is indicated below each bar (lower mesophotic—black; upper mesophotic—dark gray; shallow—light gray). Individual samples were labelled based on sample type: XM (*Xestospongia muta*), AS (*Agelas sventres*), SW (seawater), followed by sample number

At phylum level, in total 29 phyla (26 bacterial and 3 archaeal phyla) were identified (Fig. 2). Some phyla were consistently found in all *A. sventres* and *X. muta* samples: Acidobacteriota, Actinobacteriota, Chloroflexota, Gemmatimonadota, Nitrospirota, Proteobacteria (Alpha-, Gamma- and Delta-), Spirochaetota, Dadabacteria, Myxococcota and Crenarchaeota. Bacteroidota, Cyanobacteria, Entotheonellaeota and AncK6 were present in *X. muta* samples, but absent in *A. sventres* samples. Thermoplasmatota, and Marinimicrobia were only observed in seawater samples.

**Fig. 3:**
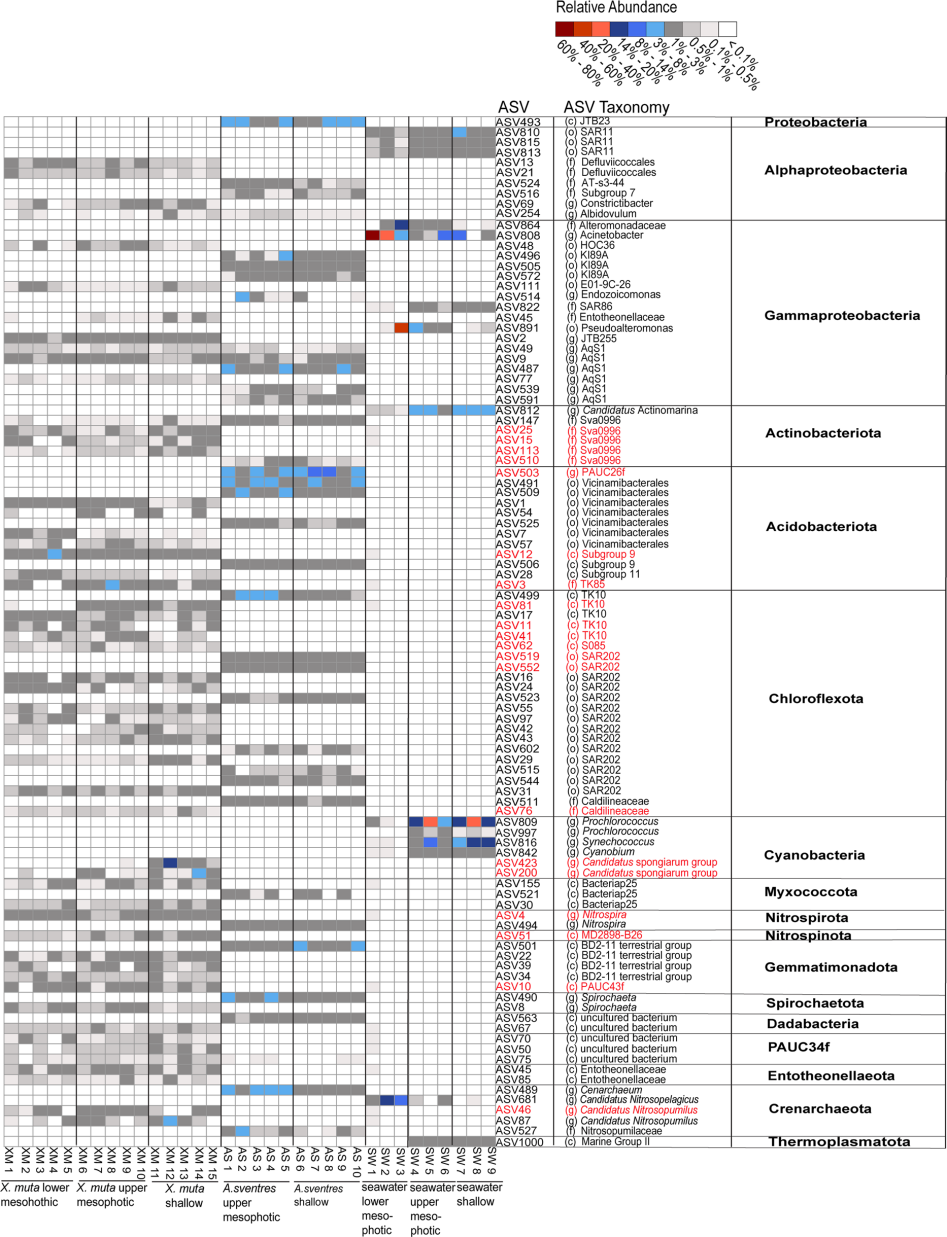
Heatmap of ASVs with average relative abundance ≥ 0.25% among all samples. ASVs were grouped at phylum level. ASVs highlighted in red were identified as “sponge-enriched” in the sponge EMP database. The letter in parentheses for ASV taxonomy indicates the lowest taxonomic rank that was obtained: c (class), o (order), f (family), g (genus)

At the ASV level, only 5 of the 100 most abundant ASVs were shared between *X. muta* and *A. sventres*: ASV254 (*Albidovulum*, Alphaproteobacteria), ASV9 and ASV49 (AqS1, uncultured Gammaproteobacteria), ASV147 (Sva0996, Actinobacteriota) and ASV75 (uncultured bacterium, PAUC34f) (Fig. 3). Furthermore, 20 (of the 100) ASVs were 100% related to sponge-enriched clusters in the sponge EMP database (Fig. 3). These belong to Actinobacteriota (Sva0996), Acidobacteriota (PAUC26f, Subgroup 9, TK85), Chloroflexota (TK10, S085, SAR202, Caldilineaceae), Cyanobacteria (*Candidatus* Synechococcus spongiarum group), Nitrospirota (*Nitrospira*), Nitrospinota (MD2898-B26), Gemmatimonadota (PAUC43f) and Crenarchaeota (*Candidatus* Nitrosopumilus).

The impact of depth was evident as indicated by differences in relative abundance of a number of predominant ASVs between the different depth zones. In shallow *X. muta*, the relative abundance of ASV200 and ASV423 both belonging to Cyanobacteria (*Candidatus* Synechococcusspongiarum_group was significantly higher than in deeper samples where ASV423 was completely absent (Fig. 3; Supplementary Table 4). Additionally, a significant decrease in relative abundance of ASV87 (Crenarchaeota, *Candidatus* Nitrosopumilus), ASV113 (Actinobacteriota, Sva0996 marine group), ASV29 (Chloroflexota, SAR202) and ASV81 (Chloroflexota, TK10) was observed from shallow to the lower mesophotic in *X. muta* (Fig. 3; Supplementary Table 4). In contrast, the acidobacteriotal ASV7 (Vicinamibacteriales) and ASV28 (subgroup 11) had a significantly higher relative abundance in the lower mesophotic compared to individuals from the upper mesophotic and the shallow waters.

In *A. sventres*, four ASVs had a significantly higher relative abundance in shallow than in specimens from the upper mesophotic: ASV503 (Acidobacteriota, PAUC26f), ASV552, ASV602 (Chloroflexota, SAR202) and ASV591 (AqS1, uncultured Gammaproteobacteria). In contrast, the relative abundance of ASV514 (Proteobacteria, *Endozoicomonas*) and ASV527 (Crenarchaeota, Nitrosopumilaceae) was significantly higher in specimens from the upper mesophotic compared to their shallow water counterparts (Fig. 3; Supplementary Table 4).

For the seawater ASVs assigned to Cyanobacteria, members of the genera *Prochlorococcus* (ASV809 and ASV997), *Synechococcus* (ASV816) and *Cyanobium* (ASV842) were present at significantly higher relative abundance in shallow and upper mesophotic samples than in the lower mesophotic seawater samples (Supplementary Table 4). These cyanobacterial ASVs in seawater were different from those observed in *X. muta*. The same trend was observed for ASV812 (Actinobacteriota, *Candidatus* Actinomarina). On the other hand, a significantly increased relative abundance in deep seawater samples was seen for ASV681 (Crenarchaeota, *Candidatus* Nitrosopelagicus) and ASV808 (Gammaproteobacteria, *Acinetobacter*) as compared to middle and shallow seawater samples.

### Antimicrobial activity of sponge tissue samples

**Fig. 4:**
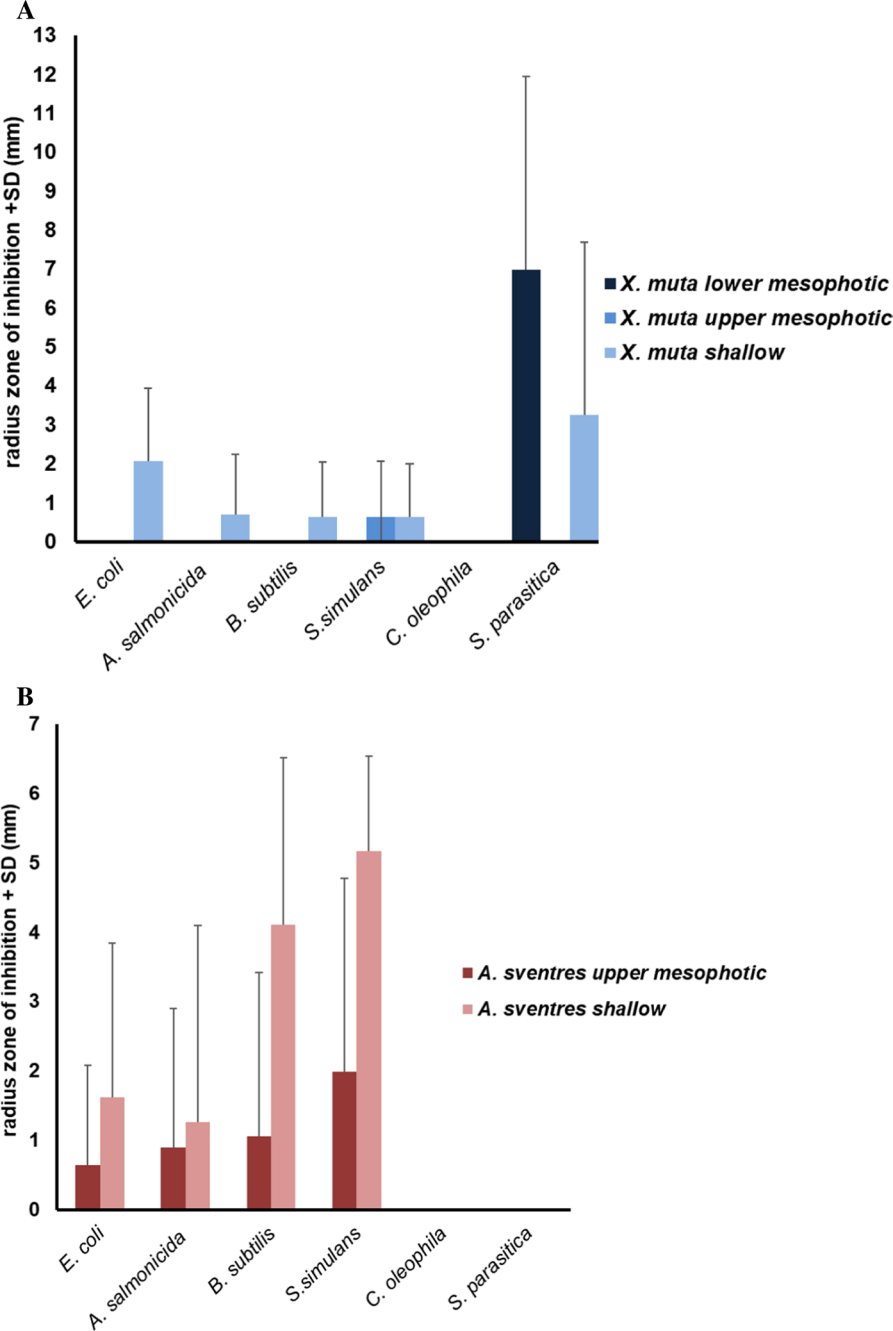
Average radius of the zone of inhibition and standard deviation of *Xestospongia muta* (**A**) and *Agelas sventres* (**B**) crude extracts against indicator strains

All 15 *X. muta* and 10 *A. sventres* crude extracts (i.e. from holobiont tissue samples) were screened for antimicrobial activity against six different indicator strains (Fig. 4A). For *X. muta*, recorded antibacterial activity came mainly from four (out of the five) shallow specimens, of which XM14 was the only crude extract that inhibited three of the four bacterial strains: *Bacillus subtilis, Staphylococcus simulans*, and *Aeromonas salmonicida* (Fig. 4A; Supplementary Table 5). Crude extracts from shallow specimens XM11, XM13 and XM15 produced small ZOI radii against *E. coli*. The only non-shallow *X. muta* specimen with antibacterial activity was XM7 (upper mesophotic depth) that was found active against *S. simulans*, whereas none of the lower mesophotic *X. muta* specimens showed antibacterial activity. All *X. muta* extracts were inactive against the yeast *C. oleophila*. In contrast, inhibition of the oomycete *Saprolegnia parasitica* was most prominent for the lower mesophotic *X. muta* specimens with two extracts with an intermediate ZOI radius (XM2 and XM4), whereas a large ZOI radius was displayed by XM3 and XM5 extracts. In addition, two shallow crude extracts of *X. muta* (XM11 and XM12) displayed a moderate inhibition against *S. parasitica*. Overall, the impact of depth was significant when the average ZOI radii of shallow *X. muta* extracts against *E. coli* were compared with those produced by the upper and lower mesophotic depth crude extracts (Tukey post hoc test, *p* = 0.03, Supplementary Table 5). Additionally, the average ZOI radius of *X. mut*a extracts against *S. parasitica* was significantly larger from specimens from the lower mesophotic compared to those from the upper mesophotic depth (Tukey post hoc test, *p* = 0.04; Supplementary Table 5). For the remaining antimicrobial activities, sampling depth did not have significant impact.

For *A. sventres*, all crude extracts from shallow specimens were active against *S. simulans* (Fig. 4B), with weak activities shown for AS7 and AS8 and moderate inhibition for AS6, AS9, and AS10 (Supplementary Table 5). Among crude extracts from specimens from the upper mesophotic zone, only AS4 (weak) and AS5 (moderate) were active against *S. simulans*. For all bacteria tested, extracts from shallow *A. sventres* individuals showed higher activity than extracts from the upper mesophotic (Fig. 4B). However, those differences between depth were never found to be significant (Supplementary Table 5). No inhibition of the yeast *C. albicans* or the oomycete *S. parasitica* was observed for *A. sventres* crude extracts.

## Discussion

In this study, we analysed the prokaryotic community composition of two sponge species, *Xestospongia muta* and *Agelas sventres*, over a depth range from near surface waters to the lower mesophotic zone (0–90 m). Both *X. muta* and *A. sventres* as well as the seawater show a shift in prokaryotic communities from the shallow to those of the upper and the lower mesophotic depth, respectively. In addition, we found changes in the activity of metabolites produced by the sponge holobionts at different water depths.

A recent study of *X. muta* revealed differences in its prokaryotic community composition between 9 and 28 m (Villegas-Plazas et al. 2018). Interestingly, those differences were only present in autumn and not in spring. Observed changes of prokaryotic composition were proposed to be triggered by differences in temperature, light, nutrient, and turbidity between seasons (Villegas-Plazas et al. 2018). Although the depth range of the *X. muta* specimens studied by Villegas-Plazas et al*.* were all in our shallow water depth, the potential environmental factors identified in that study to potentially cause differences in prokaryotic community composition have an extended gradient over larger depth. In our study, the recorded average temperature at shallow and lower mesophotic depth were 27 °C and 23 °C, respectively, which were also reported from other studies (Steinert et al. 2016; Lesser et al. 2010). Although temperature difference was evident, it is unlikely that the observed 4 °C difference between depths would be crucial to influence prokaryotic community composition (Steinert et al. 2016). For example, previous studies have shown that the elevated temperature within this range did not alter bacterial composition in sponge (Webster et al. 2008; Simister et al. 2012b). Thus, it is likely that other factors, such as irradiance, play a more pronounced effect to the observed difference of prokaryotic composition. The average irradiance at 5 m water depth in the leeward side of Curaçao was in the range of 900 µMol photons m^−2^ s^−1^ and this value reduced to 162 µMol photons m^−2^ s^−1^ (82% reduction) at 30 m depth and further decreased at 50 m to approximately 25 µMol photons m^−2^ s^−1^ (93% reduction; (Vermeij and Bak 2002)). The irradiance at lower mesophotic depth at approximately 80 m was between 10 and 19 µMol photon m^−2^ s^−1^ (98‒99% reduction from 5 m depth; (Morrow et al. 2016)). Reduced light-levels with increasing depth were reflected in the decreases in the relative abundances of photoautotrophic cyanobacteria in both seawater planktonic and *X. muta*-associated prokaryotic communities (while cyanobacteria were absent in *A sventres* both in shallow water and the mesophotic zone). Moreover, the morphological appearance of *X. muta* individuals that were observed during the sampling campaign at mesophotic depth generally displayed a paler colour (‘bleached’) compared to their shallow counterparts. Such conditions are likely related to a decline of phototrophic symbionts as also observed in individuals of sponge *Petrosia ficiformis* between well lighted and dark areas (Burgsdorf et al. 2014).Also other environmental factors than light may be involved in sponge bleaching as observed by the bleaching of the sponge *Cliona varians* forma *incrustans* at high water temperatures (Hill et al. 2016).

However, there was a marked difference between planktonic seawater cyanobacterial species and *X. muta*-associated species. Cyanobacteria in seawater were dominated by the commonly found genera *Prochlorococcus* and *Synechococcus* (Flombaum et al. 2013; Ma et al. 2009). In contrast, the most dominant cyanobacterial ASVs (ASV200 and 423) found in *X. muta* were identified as “sponge-enriched” (and different from the ones in seawater) and was identified as *Candidatus* Synechococcus spongiarum group.. Cyanobacteria are commonly found in association with HMA sponges and contribute considerably to the sponge holobiont metabolism via photosynthesis. Additionally, sponges efficiently eat cyanobacteria as part of the planktonic POM pool, specifically *Prochlorococcus* and *Synechococcus* (Yahel et al. 2003; Pile et al. 1996; Morganti et al. 2017). Consequently, the relative abundance of Cyanobacteria in sponge holobionts as well as in seawater is commonly reported to decline following a depth-dependent reduction in light availability (Morrow et al. 2016; Lesser and Slattery 2013; Lesser et al. 2020). However, the increase of inorganic nutrients and of non-cyanobacterial planktonic POM (e.g., heterotrophic bacteria, prochlorophytes) may both serve to mitigate the loss of cyanobacteria along a depth gradient (Morrow et al. 2016; Lesser and Slattery 2013; Lesser et al. 2020). In both cases, the host and the symbionts may shift to increased rates of heterotrophy for compensating the decline in irradiance, due to elevated levels of POM and inorganic nutrients, respectively (Morrow et al. 2016).

Similar to Cyanobacteria, ASVs from Chloroflexota, Acidobacteriota, Actinobacteriota, Crenarchaeota and Proteobacteria contributed significantly to differences between prokaryotic assemblages across different water depths in both sponge species. Sponges are considered as hot spots for Chloroflexota, which are especially prevalent in HMA sponges (Bayer et al. 2018; Schmitt et al. 2011). Among the most abundant Chloroflexota ASVs identified in both sponges, ASV81 (TK10) and ASV552 (SAR202) in *X. muta* and *A. sventres,* respectively, were identified as members of sponge-enriched clusters, and their relative abundances declined with depth. Although ecological functions of Chloroflexota in sponges remain unclear, in shallow habitats they may be phototrophic given that some members of this phylum possess Reaction Centre II to capture and utilize sunlight for energy (Nowicka and Kruk 2016; Ward et al. 2018). Furthermore, a metagenomics-based analysis indicated that sponge-associated Chloroflexota genomes were enriched in genes encoding glycosyl hydrolases acting on sialic acid and glycosaminoglycan suggesting their involvement in the degradation of host-derived compounds (Robbins et al. 2021). In *X. muta*, the relative abundance of the two most abundant acidobacteriotal ASVs, ASV7 (Subgroup 6) and ASV28 (Subgroup 11), increased in deep specimens (Fig. 3). Also, in *A. sventres*, acidobacteriotal ASV503 (PAUC26f) increased in relative abundance in specimens from the upper mesophotic depth. Acidobacteriota are among the prevalent heterotrophic bacterial taxa in sponges (O'connor-Sánchez et al. 2014) and are often regarded for their versatile metabolic capacities, such as nitrite and nitrate reduction, their ability to cope with disturbed or food-limited environments, and their production of exopolysaccharide (EPS), part of the DOM pool (Kielak et al. 2016). It is also plausible that Acidobacteriota might be involved in the degradation of recalcitrant organic substrates, which often accumulate in deep water habitats (Quaiser et al. 2008) or in sponge-derived polysaccharides (Robbins et al. 2021). However, due to a low number of samples, the tendency of an increase in the relative abundance of Acidobateriota with increasing depth was not found to be significant.

Actinobacteriota (Sva0996) were represented by members of sponge-enriched clusters with higher relative abundance in shallow specimens of *X. muta* (ASV113). The role of Sva0996 in sponges is unknown, however, previous studies suggested this taxon to be present at high nitrate concentrations and in high primary productivity areas (Nelson et al. 2014; Fortunato et al. 2013; Seo et al. 2017). In addition, some Actinobacteriota in sponges harbour the *tau*ABC gene encoding a taurine transporter to indicate their potential role in sulphur metabolism (Engelberts et al. 2020). The archaeal phylum of Crenarchaeota is mainly linked to ammonia oxidation and has been reported as dominant phylum in sponges both from shallow and deep water (Jackson et al. 2014; Dat et al. 2018; Zhang et al. 2014). No consistent trend was observed for the most dominant crenarchaeotal ASVs for the different sample types. While ASV87 (*Candidatus* Nitrosopumilus) is most abundant in *X. muta* in shallow specimens, the relative abundance of ASV527 (Marine group I) in *A. sventres* and ASV681 (*Candidatus Nitrosopelagicus*) in seawater was highest in deeper samples. Despite the fact that these dominant crenarchaeotal ASVs do not belong to sponge-enriched clusters, the observed trend in relative abundance may confirm that different members of this phylum are specialized to adapt to distinctive ammonia concentrations and related physical factors (temperature, light intensity, and dissolved oxygen) (Ijichi and Hamasaki 2017). Lastly, the most abundant gammaproteobacterial ASVs that changed with depth in *A. sventres*, ASV591 (AqS1) and ASV514 (*Endozoicomonas*), do not affiliate with a sponge-enriched cluster. Some possible roles assigned to members of the genus *Endozoicomonas* in sponges include antibiotic production, nitrate reduction and production of bromopyrrole as a feeding deterrent compound (Neave et al. 2016).

Sponges, as many other sessile fauna in reef habitats, need to defend themselves against biofouling, predatory organisms, and/or pathogenic bacteria (Webster 2007; Rohde et al. 2015), but this need may depend on the specific predators/competitors present in a given ecosystem (Becerro and Paul 2004). Sponge holobionts biosynthesize various antimicrobial and deterrent compounds (Helber et al. 2018). *Agelas* species are chemically well-defended sponges by producing a group of brominated-pyrrole-containing alkaloids and are unpalatable for a typical spongivorous fish such as the Bluehead wrasse, *Thalasoma bifasciatum* (Pawlik 2011; Chanas et al. 1997) and extracts of *A. sventres* individuals showed generally more diverse and stronger antibacterial activities than *X. muta* extracts. *X. muta* is also dominated by brominated compounds and feeding frequencies by parrot fishes—including *Sparisoma aurofrenarum*, *Scarus croicensis*, and *Scarus laeniopterus*—were found to increase in bleached individuals, suggesting the reduced level of cyanobacterial symbionts to be responsible for its decreased chemical defence (Dunlap and Pawlik 1998). However, no direct evidence is available at present on the shifts in production and activity of specific metabolites from the same species over a depth gradient. In this study, we observed a general trend that antimicrobial activity against the four bacterial indicator strains was higher for extracts from shallow sponges than for specimens collected at upper and lower mesophotic habitats, but this difference was generally not significant due to large intraspecific variation between biological replicates. However, it supports a recent study for a much larger depth gradient for the sponge *Geodia barretti* where the dominant secondary metabolite, barettin, completely disappeared below a depth of 1000 m (Steffen et al. 2022). Moderate to strong inhibition of *S. parasitica* was observed solely from *X. muta* crude extracts (and not *A. sventres*). Although no clear trend related to depth could be observed, the anti-*Saprolegnia* activity is an interesting observation. *Saprolegnia* spp. are fungal-like oomycetes that are parasitic to fish and fish eggs and resistant to a wide range of antifungals, making infections with *Saprolegnia* a serious threat in the aquaculture industries (Earle and Hintz 2014; Hu et al. 2013). Malachite green is the chemical most used to prevent *Saprolegnia* infections, but since the compound is toxic, also to other organisms, it has been banned world-wide (Srivastava et al. 2004; Stammati et al. 2005). Therefore, development of novel anti-*Saprolegnia* drugs is urgent (Earle and Hintz 2014; Takada et al. 2010), and anti-*Saprolegnia* metabolites from, ideally a cultivable bacterium from the sponge holobiont *X. muta* would be an interesting new lead.

## Conclusion

We investigated the impact of depth on prokaryotic community composition and antimicrobial activity associated with the tropical sponges *X. muta* and *A. sventres* from shallow water to mesophotic depth. For both species, depth had a significant impact on the associated prokaryotes with respect to different relative abundances of specific ASVs assigned to Cyanobacteria, Chloroflexota, Acidobacteriota, Actinobacteriota, Proteobacteria and Crenarchaeota. Clearly, we are just at the beginning to uncover how depth and/or depth-associated environmental conditions can cause shifts in prokaryotic communities and metabolite activity, but we show that these shifts occur. We hypothesize that changes in prokaryotic communities within the same holobiont species may therefore also change their ecological function at different depths, such as their role in chemical defence of their host. Additionally, crude extracts of shallow sponge specimens showed stronger and more diverse antibacterial activities compared to extracts from mesophotic depths, but these differences were not significant.

## Supplementary Information

Below is the link to the electronic supplementary material.

Supplementary file1 (DOCX 231 kb)

## Data Availability

Illumina MiSeq raw sequence data can be accessed via the NCBI Sequence Read Archive (SRA) ID: SRP142603 with accession numbers SRX3998987–SRX3998883. The COI gene sequences can be accessed at GenBank under the accession numbers: MH285785–MH285814. R markdown file, and the required files for 16S rRNA gene analysis can be found at https://github.com/mibwurrepo/Indraningrat-et-al.-SpongesDepthGradient2021
