# Supplementary material for: Sponge holobionts shift their prokaryotic communities and antimicrobial activity from shallow to lower mesophotic depths
Source: Antonie Van Leeuwenhoek. 2022 Aug 23;115(10):1265–83. doi: 10.1007/s10482-022-01770-4 (PMC9534810; doi:10.1007/s10482-022-01770-4)

**Supplementary Information**

**Supplementary Table 1.** Detailed overview of each sponge specimen and seawater sample arranged from deep (LM: lower mesophotic; UM: upper mesophotic) to shallow, including information regarding 16S rRNA amplicon sequence data.

| Core ID | Species | Actual Depth (m) | Depth | Number of Reads | Number of ASVs | Phylogenetic Diversity |
| --- | --- | --- | --- | --- | --- | --- |
| XM1 | *X. muta* | 66 | LM | 47230 | 200 | 19.15 |
| XM2 | *X. muta* | 86 | LM | 27517 | 165 | 17.28 |
| XM3 | *X. muta* | 72 | LM | 13666 | 180 | 18 |
| XM4 | *X. muta* | 85 | LM | 35555 | 153 | 16.16 |
| XM5 | *X. muta* | 82 | LM | 144005 | 170 | 17.11 |
| XM6 | *X. muta* | 51 | UM | 21932 | 166 | 18.25 |
| XM7 | *X. muta* | 52 | UM | 18902 | 186 | 18.63 |
| XM8 | *X. muta* | 52 | UM | 25927 | 171 | 18.11 |
| XM9 | *X. muta* | 48 | UM | 41288 | 188 | 18.02 |
| XM10 | *X. muta* | 48 | UM | 163216 | 182 | 18.38 |
| XM11 | *X. muta* | 27 | Shallow | 40656 | 160 | 17.9 |
| XM12 | *X. muta* | 27 | Shallow | 60677 | 146 | 16.93 |
| XM13 | *X. muta* | 27 | Shallow | 136515 | 165 | 17.75 |
| XM14 | *X. muta* | 27 | Shallow | 186619 | 185 | 18.51 |
| XM15 | *X. muta* | 27 | Shallow | 21179 | 159 | 17.52 |
| AS1 | *A. sventres* | 54 | UM | 104359 | 64 | 10.88 |
| AS2 | *A. sventres* | 52 | UM | 53094 | 71 | 13.06 |
| AS3 | *A. sventres* | 52 | UM | 70372 | 64 | 11.47 |
| AS4 | *A. sventres* | 54 | UM | 69361 | 76 | 11.77 |
| AS5 | *A. sventres* | 53 | UM | 19759 | 74 | 12.54 |
| AS6 | *A. sventres* | 12 | Shallow | 47923 | 68 | 12.06 |
| AS7 | *A. sventres* | 12 | Shallow | 20605 | 63 | 11.69 |
| AS8 | *A. sventres* | 12 | Shallow | 114551 | 67 | 12.34 |
| AS9 | *A. sventres* | 27 | Shallow | 144184 | 75 | 13.03 |
| AS10 | *A. sventres* | 27 | Shallow | 46515 | 77 | 12.76 |
| SW1 | seawater | 84 | LM | 111117 | 77 | 13.18 |
| SW2 | seawater | 92 | LM | 52537 | 160 | 15.28 |
| SW3 | seawater | 85 | LM | 116171 | 70 | 9.76 |
| SW4 | seawater | 50 | UM | 36066 | 137 | 14.9 |
| SW5 | seawater | 40 | UM | 68949 | 143 | 15.43 |
| SW6 | seawater | 50 | UM | 41461 | 164 | 15.76 |
| SW7 | seawater | 20 | Shallow | 49916 | 125 | 14.48 |
| SW8 | seawater | 21 | Shallow | 58323 | 117 | 14.25 |
| SW9 | seawater | 20 | Shallow | 67075 | 126 | 14.85 |

**Supplementary Table 2.** Statistical analysis on differences in phylogenetic diversity (PD) of sponge and seawater samples tested using Kruskal Wallis and Wilcoxon rank sum test based on (A) sample type and (B, C, D) depth. LM: lower mesophotic; UM: upper mesophotic for *X. muta*, *A. sventres* and seawater, respectively.

| Sample type | *A. sventres* | Seawater |
| --- | --- | --- |
| *X. muta* | 0.003 | 0.003 |
| *A. sventres* | - | 0.003 |

1. Sample type

B. Depth (*X. muta*)

| Depth | UM | Shallow |
| --- | --- | --- |
| LM | 0.015 | 0.024 |
| UM | - | 0.174 |

C. Depth (*A. sventres*)

| Depth | Shallow |
| --- | --- |
| UM | 0.007 |

D.Depth (seawater)

| Depth | UM | Shallow |
| --- | --- | --- |
| LM | 0.3 | 0.3 |
| UM | - | 0.3 |

**Supplementary Table 3.** Pairwise comparison of beta diversity on sample types and subset of sample types based on depth. LM: lower mesophotic; UM: upper mesophotic

| A | Sample type (sponge) |  |  |  |  |
| --- | --- | --- | --- | --- | --- |
|  | pairs | F.Model | R2 | p.value | p.adjusted sig |
|  | *X. muta* vs *A. sventres* | 66.00 | 0.74 | 0.00 | **0.003** |
|  | *X. muta* vs Seawater | 32.89 | 0.60 | 0.00 | **0.003** |
|  | *A. sventres* vs Seawater | 37.06 | 0.69 | 0.00 | **0.003** |
|  |  |  |  |  |  |
| B | Depth (sponge and seawater) |  |  |  |  |
|  | pairs | F.Model | R2 | p.value | p.adjusted sig |
|  | LM vs UM | 2.14 | 0.10 | 0.08 | 0.23 |
|  | LM vs shallow | 2.51 | 0.12 | 0.04 | 0.12 |
|  | UM vs shallow | 0.27 | 0.01 | 0.87 | 1 |
|  |  |  |  |  |  |
| C | Depth (*X. muta*) |  |  |  |  |
|  | pairs | F.Model | R2 | p.value | p.adjusted sig |
|  | LM vs UM | 2.78 | 0.26 | 0.01 | **0.02** |
|  | LM vs Shallow | 3.75 | 0.32 | 0.01 | **0.02** |
|  | UM vs Shallow | 1.82 | 0.19 | 0.06 | 0.17 |
|  |  |  |  |  |  |
| D | Depth (*A. sventres*) | F.Model | R2 | p.value | p.adjusted sig |
|  | UM vs Shallow | 1.786981 | 0.1825876 | 0.007 | **0.007** |
|  |  |  |  |  |  |
| E | Depth (Seawater) |  |  |  |  |
|  | pairs | F.Model | R2 | p.value | p.adjusted sig |
|  | LM vs UM | 4.924592 | 0.5518003 | 0.1 | **0.001** |
|  | LM vs Shallow | 7.989329 | 0.66637 | 0.1 | **0.001** |
|  | UM vs Shallow | 2.980473 | 0.4269729 | 0.1 | 0.7 |

**Supplementary Table 4.** The most abundant ASVs (> 0.25% relative abundance) in each sample type (*X. muta, A. sventres* and seawater) that change with depth. The highest means of abundances of ASVs in each depth category are highlighted in bold. Taxonomy is based on NG-TAX output (SILVA database 128) for Phylum and the lowest level at which it was classified . Fold difference indicate changes of abundance between different depths. LM: lower mesophotic; UM: upper mesophotic

1. *X. muta*

| **ASV** | **FDR_P** | ***X.muta* LM mean** | ***X.muta* UM mean** | ***X.muta* shallow mean** | **Taxonomy** | **Fold difference (shallow/UM)** | | | **Fold difference (shallow/LM)** |  |
| --- | --- | --- | --- | --- | --- | --- | --- | --- | --- | --- |
|  |  |  |  |  |  |  | | |  |  |
| ASV28 | 0.002 | **600.40** | 385.20 | 302.00 | Acidobacteriota, Subgroup 11 | 0.78 | | | 0.50 |  |
| ASV7 | 0.004 | **1186.20** | 150.60 | 138.40 | Acidobacteriota, Vicinamibacteriales | 0.92 | | | 0.12 |  |
| ASV113 | 0.001 | 92.20 | 296.80 | **1081.60** | Actinobacteriota , Sva0996 | 3.64 | | | 11.73 |  |
| ASV423 | 0.001 | 0.00 | 19.00 | **3299.20** | Cyanobacteria, *Candidatus* Synechococcus spongiarum group | 173.64 | | | NA |  |
| ASV200 | 0.002 | 50.00 | 324.00 | **2868.00** | Cyanobacteria, *Candidatus*  Synechococcus spongiarum group | 8.85 | | | 57.36 |  |
| ASV29 | 0.001 | 280.40 | 139.20 | **737.00** | Chloroflexota, SAR202 | 5.29 | | | 2.63 |  |
| ASV81 | 0.001 | 36.40 | 1438.00 | **2331.80** | Chloroflexota, TK10 | 1.62 | | | 64.06 |  |
| ASV45 | 0.001 | 16.60 | 151.80 | **1322.60** | Proteobacteria, Entotheonellaceae | | 8.71 | 79.67 | | |
| ASV87 | 0.03 | 382.20 | 372.00 | **1831.60** | Crenarchaeota, *Candidatus* Nitrosopumilus | 4.92 | | | 4.79 |  |

| **ASV** | **FDR_*P*** | ***A. sventres* UM mean** | ***A. sventres* shallow mean** | **Taxonomy** | **Fold differences (shallow/UM)** |
| --- | --- | --- | --- | --- | --- |
| ASV503 | 0.0005 | 3058.60 | **4445** | Acidobacteriota, PAUC26f | 1.45 |
| ASV552 | 0.0005 | 975.80 | **2416.60** | Chloroflexota, SAR202 | 2.48 |
| ASV602 | 0.0005 | 505.20 | **1231.40** | Chloroflexota, SAR202 | 2.44 |
| ASV591 | 0.0005 | 537.00 | **1126.40** | Proteobacteria, AqS1 | 2.10 |
| ASV514 | 0.02 | **1179.20** | 436.80 | Proteobacteria, *Endozoicomonas* | 0.37 |
| ASV527 | 0.001 | **1322.8** | 559.6 | Crenarchaeota, Nitrosopumilaceae | 0.42 |

B. *A. sventres*

C. seawater

| **ASV** | **FDR_P** | **seawater LM mean** | **seawater UM mean** | **seawater shallow mean** | **Taxonomy** | **Fold difference (shallow/UM)** | **Fold difference (shallow/LM)** |
| --- | --- | --- | --- | --- | --- | --- | --- |
| ASV812 | 0.001 | 553.67 | 2490.33 | **3401.00** | Actinobacteriota, *Candidatus* Actinomarina | 1.37 | 6.14 |
| ASV809 | 0.001 | 617.67 | 7299.00 | **11181.67** | Cyanobacteria, Prochlorococcus | 1.53 | 18.10 |
| ASV816 | 0.001 | 193.67 | 3156.33 | **7847.33** | Cyanobacteria, Synechococcus | 2.49 | 40.52 |
| ASV842 | 0.001 | 91.67 | **1008.67** | 961.00 | Cyanobacteria, Cyanobium | 0.95 | 10.48 |
| ASV997 | 0.001 | 0.00 | **925.33** | 355.67 | Cyanobacteria, Prochlorococcus | 0.38 | N/A |
| ASV808 | 0.001 | **34672.33** | 2481.33 | 2004.33 | Proteobacteria, Acinetobacter | 0.81 | 0.06 |
| ASV681 | 0.002 | **7519.67** | 633.67 | 22.67 | Crenarchaeota, *Candidatus* Nitrosopelagicus | 0.036 | 0.003 |

**Supplementary Table 5.** Statistical test on the radius zone of inhibition (ZOI) of sponge crude extracts against microbial indicator strains. Panel A: Comparison of ZOI radii of sponge extracts of *X. muta* from different depths. Panel B: Comparison of ZOI radii of sponge extracts of *A. sventres* from different depths. In both tables, recorded ZOIs for each crude extract are provided, along with average inhibition and standard deviation per depth category. Analysis of variance (anova) was applied with p-values <0.05 being indicative of significant differences (highlighted in bold). Subsequently, only values found significant were tested using the Tukey post hoc test to determine which pair-wise comparison of sponge crude extracts gave a statistically significant result (highlighted in bold). Non-significant anova results were not tested in Tukey post hoc test (N/A, not applicable). ZOI radii were grouped into four categories, namely weak (0 -5 mm), moderate (5-10 mm), strong (> 10-20 mm) and very strong (> 20 mm). LM: lower mesophotic; UM: upper mesophotic

A. *X. muta*

| radius zone of inhibition (mm) | | | | | | |  |
| --- | --- | --- | --- | --- | --- | --- | --- |
|  | Sponge extracts | *E. coli* | *A. salmo-nicida* | *B. subtilis* | *S. simulans* | *C. oleo-phila* | *S. para-sitica* |
| LM | XM1 | 0 | 0 | 0 | 0 | 0 | 0 |
| LM | XM2 | 0 | 0 | 0 | 0 | 0 | 5.55 |
| LM | XM3 | 0 | 0 | 0 | 0 | 0 | 13.43 |
| LM | XM4 | 0 | 0 | 0 | 0 | 0 | 6.51 |
| LM | XM5 | 0 | 0 | 0 | 0 | 0 | 9.38 |
| mean inhibition and standard deviation | | 0±0 | 0±0 | 0±0 | 0±0 | 0±0 | 6.97±4.96 |
| UM | XM6 | 0 | 0 | 0 | 0 | 0 | 0 |
| UM | XM7 | 0 | 0 | 0 | 3.2 | 0 | 0 |
| UM | XM8 | 0 | 0 | 0 | 0 | 0 | 0 |
| UM | XM9 | 0 | 0 | 0 | 0 | 0 | 0 |
| UM | XM10 | 0 | 0 | 0 | 0 | 0 | 0 |
| mean inhibition and standard deviation | | 0±0 | 0±0 | 0±0 | 0.64±1.43 | 0±0 | 0±0 |
| shallow | XM11 | 3.72 | 0 | 0 | 0 | 0 | 8.14 |
| shallow | XM12 | 0 | 0 | 0 | 0 | 0 | 8.09 |
| shallow | XM13 | 3.27 | 0 | 0 | 0 | 0 | 0 |
| shallow | XM14 | 0 | 3.47 | 3.14 | 3.1 | 0 | 0 |
| shallow | XM15 | 3.28 | 0 | 0 | 0 | 0 | 0 |
| mean inhibition and standard deviation | | 2.05±1.88 | 0.69±1.55 | 0.63±1.40 | 0.62±1.39 | 0±0 | 3.25±4.44 |
| ANOVA (p-value) | | **0.02** | 0.40 | 0.40 | 0.60 | N/A | **0.04** |
|  |  |  |  |  |  |  |  |
|  |  |  |  |  |  |  |  |
| Tukey Post hoc test | pvalue_LM_UM | 1 | 1 | 1 | 1 | N/A | **0.04** |
|  | pvalue_LM_shallow | **0.03** | 0.5 | 0.5 | 0.5 | N/A | 0.3 |
|  | pvalue_UM_shallow | **0.03** | 0.5 | 0.5 | 0.5 | N/A | 0.4 |

| radius zone of inhibition (mm) | | | | | | |  |
| --- | --- | --- | --- | --- | --- | --- | --- |
| Depths | Sponge extracts | *E. coli* | *A. salmonicida* | *B. subtilis* | *S.simulans* | *S. parasitica* | *C. oleophila* |
| UM | AS1 | 0 | 0 | 0 | 0 | 0 | 0 |
| UM | AS2 | 0 | 0 | 0 | 0 | 0 | 0 |
| UM | AS3 | 0 | 0 | 0 | 0 | 0 | 0 |
| UM | AS4 | 0 | 0 | 0 | 4.07 | 0 | 0 |
| UM | AS5 | 3.22 | 4.47 | 5.27 | 5.86 | 0 | 0 |
| mean inhibition and standard deviation | | 0.64±1.44 | 0.89±2.00 | 1.05±2.36 | 1.99±2.79 | 0±0 | 0±0 |
| shallow | AS6 | 4.15 | 0 | 5.59 | 6.86 | 0 | 0 |
| shallow | AS7 | 0 | 0 | 0 | 3.15 | 0 | 0 |
| shallow | AS8 | 0 | 0 | 4.01 | 4.68 | 0 | 0 |
| shallow | AS9 | 3.96 | 6.32 | 5.93 | 5.5 | 0 | 0 |
| shallow | AS10 | 0 | 0 | 5 | 5.63 | 0 | 0 |
| mean inhibition and standard deviation | | 1.62±2.22 | 1.26±2.83 | 4.11±2.41 | 5.16±1.37 | 0±0 | 0±0 |
| ANOVA (p-value) | pvalue_UM_shallow | 0.4 | 0.8 | 0.08 | 0.05 | N/A | N/A |
|  |  |  |  |  |  |  |  |

B. *A. sventres*


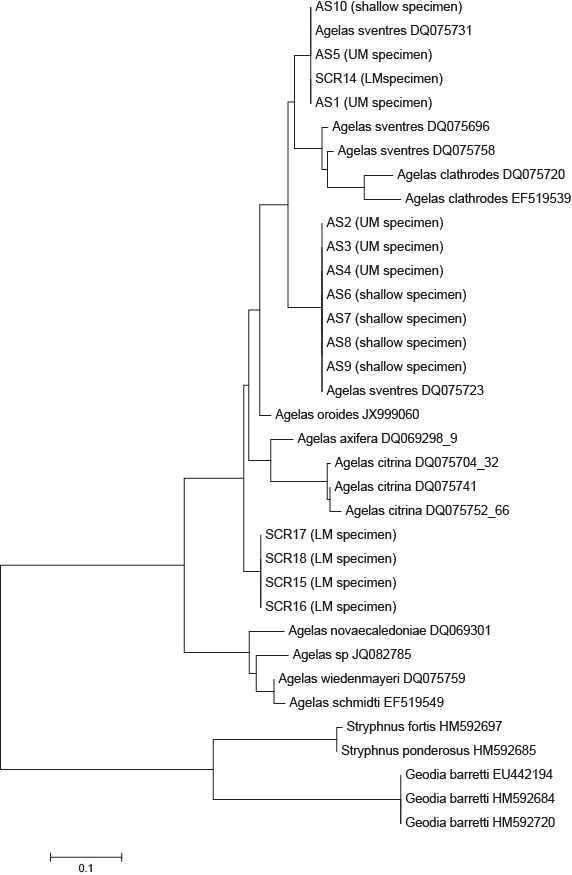
**Suplementary Figure 1.** (A). Phylogenetic tree constructed with the maximum likelihood algorithm with 500 bootstrap replications and Nearest-Neighbor-Interchange (NNI) for ML Heuristic of *X. muta* specimens and (B) *A. sventres* specimens based on the COI gene. The number (0.1) below the reference bar indicates percentage distance of sequence. LM: lower mesophotic; UM: upper mesophotic


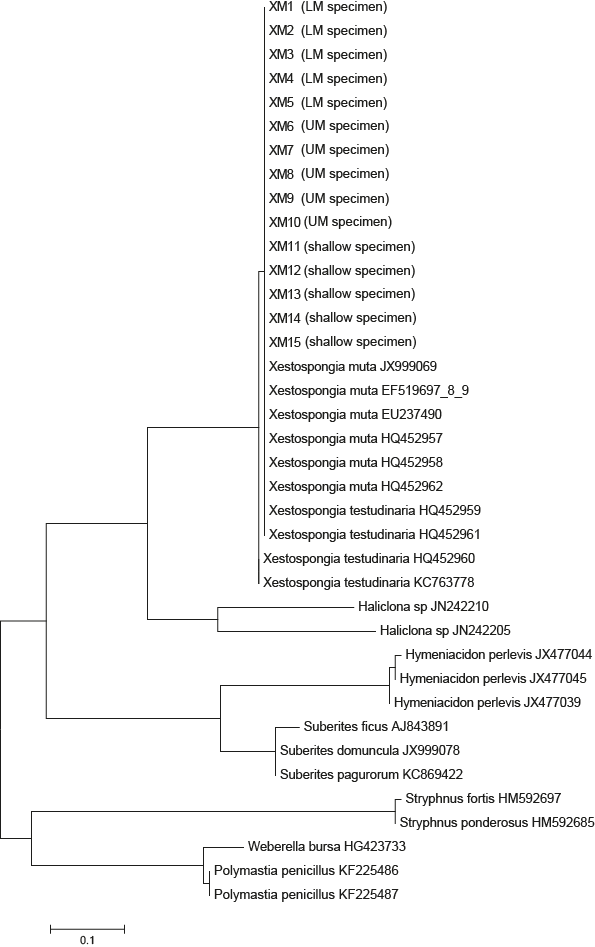


B

A

**Supplementary Figure 2.** Phylogenetic diversity (PD) of prokaryotic communities in *X. muta*, *A. sventres* and seawater at different depths.


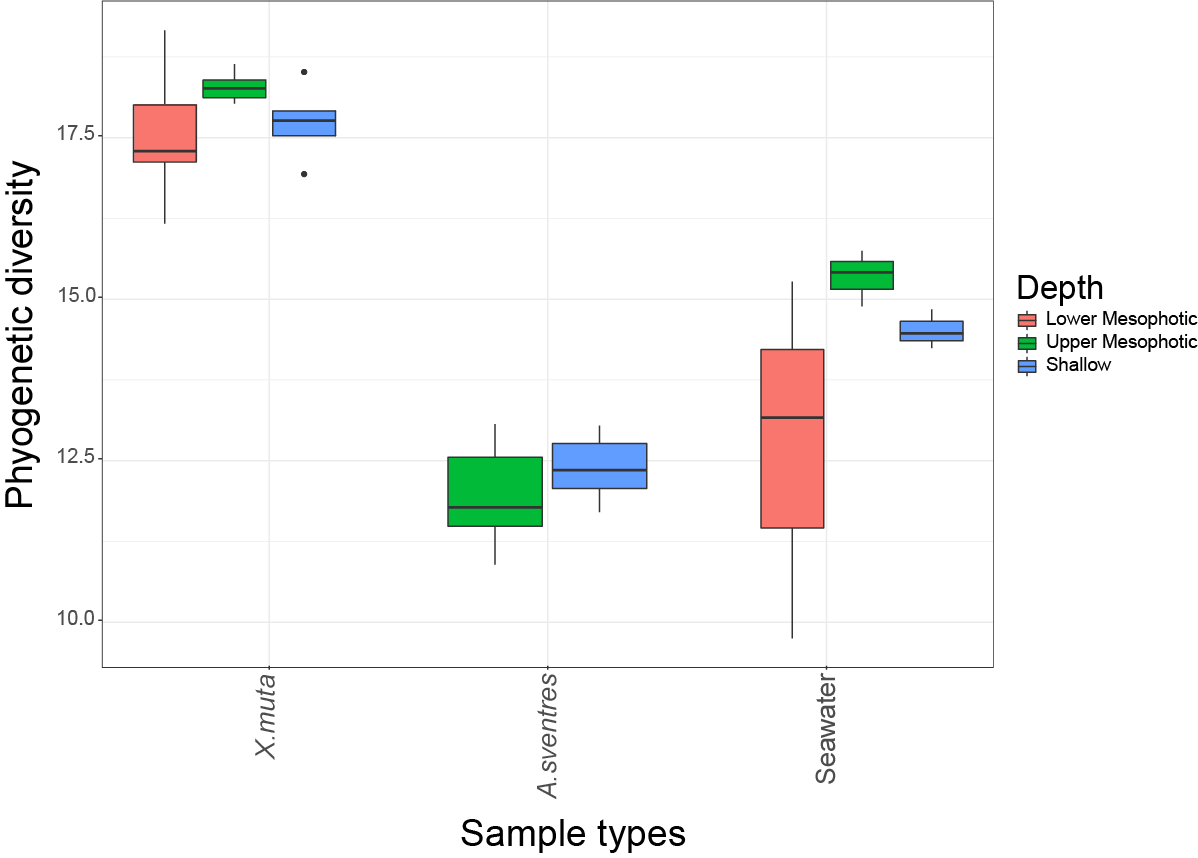

Supplement: Supplementary file 1 — Supplementary file1 (DOCX 231 kb) [file 10482_2022_1770_MOESM1_ESM.docx]
